# What influences the performance of carbon emissions in China?—Research on the inter-provincial carbon emissions’ conditional configuration impacts

**DOI:** 10.1371/journal.pone.0293763

**Published:** 2024-04-10

**Authors:** Weidong Chen, Dongli Li, Quanling Cai, Kaisheng Di, Caiping Liu, Mingxing Wang

**Affiliations:** 1 Department of Management and Economics, Tianjin University, Tianjin, China; 2 College of Chunming, Hainan University, Haikou, China; 3 College of Politics and Public Administration, Qinghai Minzu University, Xining, China; 4 College of Finance and Economics, Qinghai University, Xining, China; Shandong University of Science and Technology, CHINA

## Abstract

The severe global warming issue currently threatens humans’ existence and development. Countries and international organizations have effectively implemented policies to reduce carbon emissions and investigate low-carbon growth strategies. Reducing carbon emissions is a hot topic that academics and government policy-making departments are concerned about.Through necessary condition analysis (NCA) and fuzzy set qualitative comparative analysis(fsQCA), this paper investigates local governments’ configuration linkage effect and path choice to improve carbon emission performance from six dimensions: energy consumption, industrial structure, technological innovation, government support, economic development, and demographic factors. The research findings include the following: (1) Individual condition does not represent necessary conditions for the government’s carbon performance. Among the two sets of second-order equivalence configurations(S and Q) (five high-level carbon performance configurations), those dominated by economic development or low energy consumption can produce high-level carbon performance. Therefore, the six antecedent conditions dimensions work together to explain how the government can create high levels of carbon performance. (2)According to the regional comparison, China’s eastern, central, and western regions exhibit similarities and differences in the driving forces behind high carbon emission performance. All three regions can demonstrate carbon emission performance when all the factors are combined. However, when constrained by the conditions of each region’s resource endowment, the eastern region emphasizes the advantage of economic and technological innovation, the central region favors government support and demographic factors, and the western region prefers upgrading industrial structure based on a specific level of economic development.

## Introduction

Numerous greenhouse gases have been released due to increased global industrialization and the overuse of non-renewable energy sources [1]. The increase in global temperature causes species extinction, droughts, floods, forest fires, ocean acidification, and sea level rise, among other ecological and societal problems [2, 3]. Issues arising from climate change have significantly impacted the shifting patterns of global politics, economy, society, and environment [4, 5]. In response to the environmental issues caused by rising global greenhouse gas concentrations, countries have reached a consensus to reduce carbon dioxide emissions since the signing of the Paris Agreement in 2015 [6]. As of the end of 2021, 136 countries around the globe have put forward "carbon neutrality" targets (By the end of 2021, 136 countries around the world have proposed "carbon neutral" targets [7]. China is the world’s largest CO2 emitter and is critical in mitigating global climate change. China’s "3060" carbon peaking and carbon neutrality targets are a vital part of China’s commitment to fulfilling the Paris Agreement [8].

In recent years, the Chinese government has recently implemented several policies to encourage adopting low-carbon growth. The National Plan for Addressing Climate Change (2014–2020) released by China’s National Development and Reform Commission (NDRC)explicitly proposed to reduce carbon emissions by 40–45% per unit of GDP (carbon intensity) by 2020 through measures like adjusting the industrial structure, optimizing the energy structure.Secondly, In addition, the Chinese government has also adopted several low-carbon emission reduction measures to ensure energy conservation and emission reduction, including the creation of a carbon emission market [9], low-carbon pilot cities [10], energy restructuring [11], industrial restructuring [12], carbon finance [13], and laws and regulations related to low-carbon development [14].However, as a developing country, China’s economic development is still the main task of national development. Against this background, balancing the coordinated development of low-carbon emission reduction and the economy is a hot issue of concern to many social and economic scholars.Carbon emission performance description indicates the relationship between carbon emissions and economic development, and the common evaluation methods include single-factor and full-factor [15]. The single-factor indicators include carbon intensity [16–18] and carbon productivity [19]. Carbon intensity (CI) is an effective indicator for governments worldwide to balance economic growth and environmental concerns [20]. It is often defined as the ratio of carbon emissions to gross domestic product (GDP) [21].Total factor carbon performance, also known as total factor carbon productivity, is the relationship between input factors’ desired and actual output under particular economic and technological circumstances [22].The article refers to [16, 17] chose the single factor carbon intensity as the measure of carbon emission performance because it focuses on the internal mechanism of the linkage effect of each condition under the equilibrium result of carbon emission and economic development. The input-output mechanism between the condition and the result is not obvious.Current study on carbon emission performance by domestic and international academics concentrates on result accounting and influencing factors. There are few studies on the various elements’ linkage effect and the influence mechanism of multiple factors influencing carbon emission performance.Therefore, this paper proposes a holistic analysis framework that affects the government’s carbon emission performance from the perspective of multiple causal concurrent configurations and explores the path mechanism that affects the government’s carbon emission performance based on the national policy objectives and the path selection mechanism of low-carbon emission reduction by domestic and foreign scholars.Based on the availability of data, eighteen quantitative indicators were chosen from six categories based on available data, including energy consumption, industrial structure, technological innovation, government support, economic development,and demographic factors. Using 30 provincial-level administrative regions of China (excluding Tibet) as a case study, we explored the necessary condition analysis (NCA) and fuzzy set qualitative comparative analysis (fs/QCA) to analyze the necessary and sufficient [23]. Furthermore, fs/QCA and NCA examine the two kinds of causality, necessary and sufficient, to address the issues below:

1. What condition configuration impacts the inter-provincial government emission performance?
2. To what degree are these conditions required for high carbon emission performance?
3. Is there any distinction in government carbon emission performance in China’s eastern, central, and western regions?

**Fig 1:**
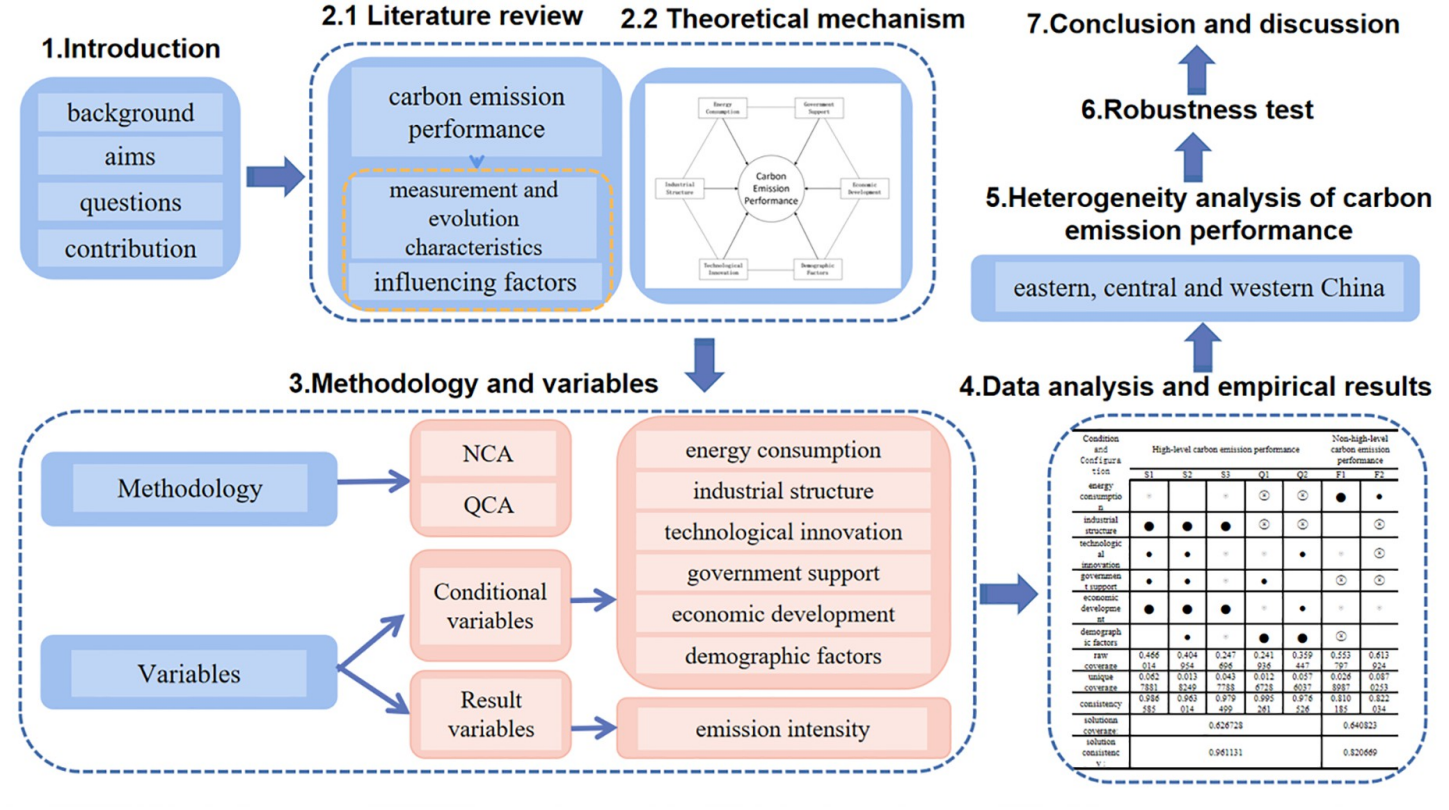
Structural diagram of this article.

The remainder of this essay is organized as follows: The section on research review and framework describes the literature review and presents the research’s theoretical framework based on a review of the past. The section on data construction and research methods introduces data construction and research methods, explains the meaning of each variable, and details the data sources. The section on data analysis and empirical results summarizes and analyzes the necessity analysis of individual results and configuration. The next section introduces the differentiation path of carbon emission performance in eastern, central and western China, and the robustness test is covered in Section robustness test. Finally, The section on Conclusion and discussion provides conclusions and policy implications—the structural diagram of the article shown in Fig 1.

## Research review and framework

### Practical experience and research progress of carbon emission performance

The balance between energy conservation, emission reduction, and economic growth is one of the hot topics that many academics are concerned about under the low-carbon development trend.Currently, domestic and foreign scholars mainly study carbon emission performance from two perspectives.(1)The measurement and evolution characteristics of carbon emission performance. First of all, in the calculation of carbon intensity research, the ratio of regional carbon emissions to GDP is used as the study’s primary direct measure of carbon intensity. Undeniably, the carbon intensity (CO2 / GDP) has decreased over the past few decades in all countries. However, some people think that this has not resulted in a reduction in global CO2 emissions because its effects have been countered by GDP development., Goldemberg [21] quantified the reduction of carbon dioxide emissions in 14 countries and the European Union (EU) between 2000 and 2018. It is found that most countries have a 10–40% reduction in carbon emissions, which also shows that carbon intensity is effective as an indicator to measure carbon emission performance.Yang & Su’s [24] analysis of the global carbon intensity in 2014 and the global carbon intensity of exports in 44 regions found that exports globally increased carbon intensity by 7.2%, particularly exports of carbon-intensive sectors. (electronics, computers, and communication technology) will increase global and national carbon emission intensity. Sun [25] examined how the intensity of India’s different economic sectors’ carbon emissions changed between 1995–2009. The findings demonstrate that EGW (electricity, gas, and water supply), responsible for 60% of CO2 emissions from the secondary industry, is the most well-known high-carbon sector from a production viewpoint.Second, for total factor carbon productivity measurement, researchers have measured carbon emissions in industry, agriculture [26, 27], the construction industry [22], the urban logistics industry [28], and pharmaceutical industry [29] based on the mechanism of input and output performance.Finally, the spatial characteristics and evolution trends of carbon emission performance on a large scale are considered to be the basis for exploring carbon-neutral measures in different regions. Zhao [20]examined the spatial and temporal variability of carbon intensity levels related to energy consumption, the spatial heterogeneity of its driving forces, and the intensity of its influencing factors. Based on the carbon emission performance of 191 prefecture-level cities in China from 1997 to 2017, Wang [15] examined the evolutionary characteristics of urban carbon emission performance in terms of overall spatial and temporal evolution, differences at the regional and city scales, and variations in the classification clusters of urban carbon emission performance.

(2)The influencing factors of carbon emission performance. The influencing factors of carbon emission performance are frequently inseparable from carbon emission reduction measures and green economic development as a measure of the connection between low carbon and economic development. According to their traits, the influencing factors are divided into internal and external influencing factors. The establishment of low-carbon cities, the use of clean energy, industrial structure, industrial transformation, and upgrading, population size, urbanization rate, GDP, and GDP per capita are examples of external influencing factors. On the other hand, internal influencing factors directly affect changes in carbon emissions and have specific statistical characteristics.Due to its immediate impact on carbon emissions, the endogeneity factor is a major research topic for many academics.Zhao [20] used geographically weighted regression to conclude that energy structure has the greatest impact on carbon intensity, followed by demographic factors like total population and urbanization rate, economic factors like industrial structure and GDP per capita, as well as foreign investment and openness to foreign trade, all of which have varying degrees of impact. They were building in urban development zones, attracting foreign funding, and expanding internationally.Chang [30] and Lu [31] concluded that the structure of energy consumption significantly impacts carbon emission performance and that optimizing the energy structure to lower carbon intensity is a practical strategy for creating low-carbon city development models. Their research on the connection between renewable energy and CO2 emission intensity in the nations with the highest CO2 emission intensities between 2000 and 2015. Mirziyoyeva & Salahodjaev [32] discovered that renewable energy has a sizable detrimental impact on CO2 emissions. Due to China’s rapid economic development, there has been an uptick in urbanization and industrial upgrading, which has increased energy consumption and carbon dioxide emissions.Zheng [33]studied that carbon emissions in China have stabilized since 2012, largely due to increased energy efficiency and upgraded industrial and energy structures. However, heterogeneity in the combined effects of these two drivers on carbon emissions across different regions exists. Zhang [34] examined the influences of economic growth, industrial structure, and urbanization on China’s carbon emissions intensity between 1978 and 2011. The findings indicated that the increase in the share of tertiary industry and economic growth had a significant impact on reducing carbon emission intensity, and the rate of urbanization may result in an increase in carbon emission intensity.Yang [35] investigated how Beijing’s population-related variables affected carbon emissions between 1984 and 2012. The findings indicated that urbanization was the primary cause and that the population’s age composition alteration significantly reduced carbon emissions. The researchers Su [36] and Huang & Chen [37] concluded that R&D spending can boost technological innovation as a means to optimize energy structure, lower energy intensity, and achieve green development, which can lower carbon dioxide (CO2) emissions.

Additionally, some academics believe that implementing a few government-led external policies will affect the efficacy of carbon emissions.Lin & Zhou [38] argued that Internet development could improve carbon emission performance by promoting the structural upgrading of industries and technology diffusion. According to Song [39], foreign direct investment is a major contributor to carbon emissions and a key driver of global economic growth. The research found that, overall, foreign direct investment has a less negative impact on carbon emission performance than it does positive effects.Wang [40] analyzed the effect of trade openness on carbon intensity in 104 countries/regions during 2000–2014 through a panel threshold model. They found that the effect of trade openness on carbon intensity was negatively correlated with foreign direct investment as the threshold variable.Some Chinese scholars have conducted policy experiments through a double difference approach and concluded that the construction of urban development zones [41] and the establishment of carbon markets [42] could effectively improve carbon performance and the intrinsic mechanism is that development zones influence urban carbon performance by increasing urban GDP and reducing urban carbon emissions. The carbon market is an intermediate channel to promote improving carbon emission performance in China through technological innovation. The government must invest in R&D emission reduction technology support and encourage green preferences in R&D investment.

Current research is abundant on the mechanisms that affect carbon emission performance. However, little work has been done to explain which path to take and how carbon emission performance varies by area.Combining the reviews mentioned above, we consider that the current research presents the following flaws:(1) Although existing studies have provided rich measures and influencing factors for research on carbon emission performance, it is challenging to offer enough theoretical support for choosing the carbon emission performance path. (2) The factors influencing carbon emission performance have historically interacted rather than existing independently. The existing literature’s unified symmetric relationship assumption between independent and dependent variables has restricted the path selection for improving carbon emission theory performance. (3)Existing studies have yet to fully analyze the complex internal mechanisms influencing carbon performance. Little attention has been paid to the mechanism underlying the antecedent conditions creating the configuration of high-level performance.

Based on previous research, the possible marginal contributions of this paper are: (1) The article tries to explore the effect of the combination of the effect of different influencing factors on carbon emission performance from the group state perspective, distinguishing from the analysis of the effect of a single element, through the analysis of the group state results of high carbon emission performance, which can provide specific theoretical support for the local government to enhance the choice of carbon emission performance path. (2) In line with the actual situation, multi-factors are not a single factor affecting carbon emission performance, and the study of the linkage effect between multi-factors further enriches the complex mechanism between the influencing factors of carbon emission performance.

#### Carbon emission performance research framework

**Fig 2:**
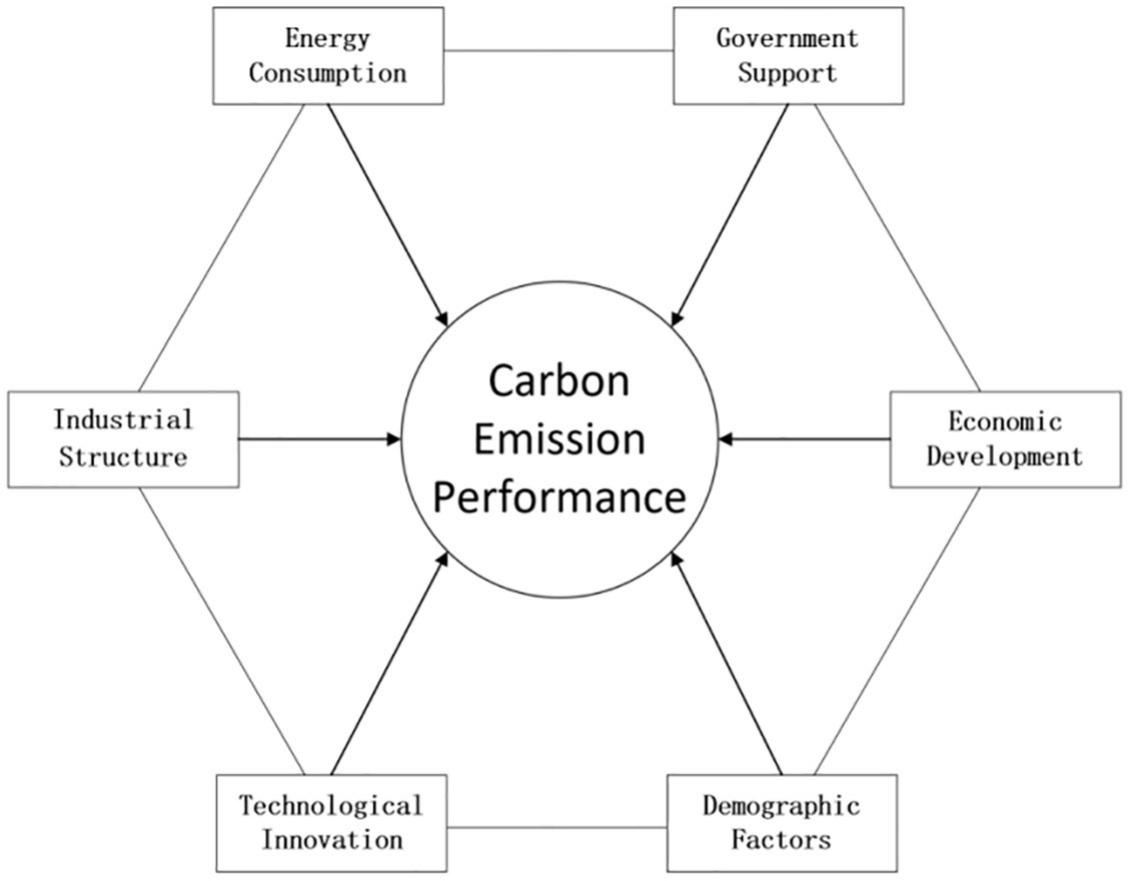
Carbon emission performance mechanism.

Based on scholars’ analyses of the factors influencing carbon emission performance (carbon intensity),the paper makes an effort to use the qualitative comparison (QCA) method, choose 30 provincial data in 2019, and explore the theoretical framework model that affects carbon emission performance from six dimensions of energy consumption, industrial structure, technological innovation, government support, economic development, and demographic factors (Fig 2), in order to explore the linkage effects among the six antecedent conditions.

#### Energy consumption

This condition contains three indicators: total energy consumption, energy intensity, and energy mix (coal share). Energy is necessary for satiating basic needs and achieving economic development objectives, and production techniques that rely on fossil fuels increase carbon emissions [43]. Using of fossil fuels, such as coal, has been one of the significant contributors to haze and carbon emissions. China consumes 70% of the world’s coal, according to data from the National Bureau of Statistics and the National Energy Statistics Bureau of China. Even though energy intensity is declining globally, China’s energy consumption remains a significant source of carbon emissions. Generally, there has been a long-standing correlation between energy consumption, GDP, and carbon emissions [44]. Energy consumption is an essential component of contemporary economic development, and the amount of energy consumed is directly related to carbon dioxide (CO2) emissions, which is an essential factor in shaping the pattern of changes in global carbon intensity (carbon emission performance) [45]. The energy mix determines the share of coal in energy consumption. An energy mix with a high coal share is more likely to produce high carbon emissions [46].Energy intensity measures a nation or region’s energy effectiveness by calculating the energy consumed per unit of GDP [47]). One of the critical strategies for reducing carbon emissions and global energy consumption is to decrease energy intensity [48]). As a high-energy consumer, China’s energy use raises carbon intensity through increased carbon emissions,suppressing carbon emission performance. Asymmetric causal configuration strongly supports this condition as a factor negatively affecting carbon emission performance.

#### Industrial structure

It includes the rationalization index of industrial structure, the advanced index of industrial structure, and the proportion of tertiary industry in GDP.China’s economic development has entered a new normal. Within the framework of supply-side structural reform, the upgrading and adjustment of industrial structures have evolved into the foundation of economic development [49–51]. The industrial structure is a key factor affecting overall carbon emissions or energy consumption [52]. Rationalization and advancement of industrial structure is the primary indicator of industrial transformation and upgrading [53]. Studies have shown that the optimization and upgrading of industrial structure can effectively curb carbon emissions and enhance regional carbon emission performance [54], especially the promotion of rationalization and advancement of industrial structure in resource-endowed regions will help to curb carbon dioxide emissions and enhance the efficiency of carbon emissions [55]. The proportion of tertiary industry in GDP reflects the degree of inclination of economic structure towards tertiary industry and is also an indicator of industrial transformation and upgrading.Researches indicate that the proportion of secondary industry has a significant positive impact on carbon emissions and energy use [56, 57], whereas an increase in the proportion of tertiary industry can significantly reduce carbon emissions [58].

#### Technological innovation

It includes three indicators: R & D investment in science and technology, the full-time equivalent of R & D personnel in industrial enterprises above the designated size, and the number of green patents. Technological advancement can optimize the energy mix for specific areas and sectors, encourage the use of renewable energy sources, and significantly lower CO2 emissions [36, 37].Investment in corporate R&D can also lower carbon emissions and improve environmental performance, but the effect is small [59, 60].The sustainable development objectives established by the United Nations include clean technologies. Future green technology innovation and renewable energy use geared toward clean energy will be the primary drivers of decreased energy use and carbon emissions [61]. According to the International Energy Agency’s (2013) 450 scenario, green technologies, such as increasing renewable energy sources and boosting energy efficiency, are anticipated to be the most effective at reducing global warming by more than 60%. Innovation in green technology is, therefore, essential for all nations around the globe.

#### Government support

Three indicators are selected: government green development attention, government integrated government service ability and Chinese government transparency index.Government attention is the focus and direction of governmental governance resources on a particular issue or a particular type of affairs, and the allocation of governmental attention is a significant influence on governmental decision-making behavior and the formulation of public policy, which is connected to the enhancement of the effectiveness of public policy and public governance [62]. The focus of attention determines the decisions made by policymakers, and the limited nature of attention and shifts in attention will affect both stable and abrupt changes in policy [63–65]. Liu [66] argued that there is a positive synergy between governmental attention and environmental and social feedback and that increasing governmental attention to the environment will effectively reduce environmental pollution. In contrast, an increase in governmental attention, represented by regional leaders, may have a facilitating effect on the performance of regional carbon emissions by influencing the intensity of regional R&D investment and the level of marketization [67].Government information disclosure is a crucial component of government governance activities. Government performance management is based on information transparency, fair and open evaluation, and ensuring the management process is fully presented and disclosed to the public. It allows for better public participation, which is suitable for environmental performance and contributes to the government’s carbon performance [68, 69]. E-government is an important symbol and means of reforming the administrative capacity of Governments and plays a significant role in improving governance capacity, promoting economic transformation, and enhancing environmental management performance [70]. On the one hand, e-government is an essential part of the government’s digitalization process, which reduces the waste of resources compared to the traditional mode of handling government affairs, and digitalization has a good synergy effect on energy saving and emission reduction [71]. On the other hand, e-government dramatically improves the efficiency of the government’s work, and by enhancing the credibility of the government and its execution and increasing the public’s enthusiasm to participate in governmental work, it can improve the efficiency of government management and environmental performance. On the other hand, e-government dramatically improves government work efficiency, enhances public participation in government work by strengthening government credibility and implementation, and improves government management efficiency and environmental performance [72].

#### Economic development

It includes three indicators: gross domestic product (GDP), per capita regional GDP, and digital financial inclusion index. Economic growth is a crucial metric for measuring carbon emission success. China has made significant economic strides, but these successes have been followed by rapid industrialization and urbanization marked by high energy consumption, high pollution, and high emissions. There are many interactions between this rapid economic growth and high levels of carbon emissions pollution. While GDP per capita represents the degree of economic development, the gross domestic product (GDP) is the ultimate indicator of economic development. Digital financial inclusion has been made possible by combining conventional finance and advanced digital technologies [73], which can reduce carbon intensity (improves carbon emission performance) by optimizing industrial structure, promoting green technology, and showing spillover effects in space [74].

#### Demographic factors

It includes three indicators: year-end resident population, urbanization rate and urban population density.The internal mechanism underlying the impact of demographic variables on carbon emissions is complicated. Urbanization is one of them. From the perspective of total emissions, human activities result in city carbon emissions [75]. The number of people residing in cities also affects CO2 emissions and economic activities [76, 77], and the growth of the urban population increases the total carbon emissions [56].Although an increase in urban population size results in more CO2 emissions overall [78], per-capita emissions have decreased due to the ability of urban population growth to advance technology, increase the effectiveness of using public facilities, and lower CO2 emissions [79, 80]. According to Hong’s research the critical urban population size is one million [81]. When fewer than one million people live in an urban area, growing urban density can help reduce carbon emissions. In contrast, greater urban density results in higher carbon emissions when a city’s population surpasses one million. As a result, demand for urban carbon emissions is driven by population, and there is a strong correlation between urban population and urban carbon emissions.

## Data construction and research methods

### Data construction and collection

#### Result variables

The ratio of carbon emissions to the province’s total GDP is used in the article as a measure of carbon emission performance. The greater the carbon emission intensity, the worse the carbon emission performance is depicted. The province GDP data is sourced from the National Bureau of Statistics, and the data on carbon emissions are taken from the China Stock Market & Accounting Research Database (CSMAR).

#### Conditional variables

1. *Energy consumption*. Energy consumption has a decisive influence on carbon emission intensity, among which coal, a fossil energy source, occupies a considerable proportion of energy consumption and is also the main factor affecting energy carbon emission. The article selects three indicators, namely energy intensity, total energy consumption, and the proportion of coal in energy consumption, to comprehensively measure the energy consumption condition variables. The data on total energy consumption are obtained from the CSMAR database, energy intensity is calculated based on the ratio of total consumption to provincial GDP, and coal share is calculated based on the National Energy Statistics Bureau of China.
2. *Industrial structure*. Three indexes are selected: the index of industrial structure rationalization and advanced index, and the proportion of tertiary industry in GDP. The industrial structure rationalization index is measured by the calculation method of the Taylor index, which reflects the deviation of the output value structure and the employment structure of the three major industries in China. If the index is 0, the industrial structure is at equilibrium. If it is not 0, the industrial structure deviates from the equilibrium state, and the industrial structure is unreasonable [53]. The index of industrial advance refers to Gan [53], which adopts the proportion of tertiary industry to secondary industry, and the value is increasing, which means that the economy is advancing in the direction of service, and the industrial structure is upgrading. The data of tertiary industry share are obtained from the National Bureau of Statistics of China.
3. *Technological innovation*. Technological innovation can enhance green and clean technology through research and development, which is important in reducing carbon dioxide emissions. The investment and number of scientific and technological research and development reflect the regional level of science and technology and the ability to transform scientific research results into technology and products. This data comes from the National Bureau of Statistics of China. The number of patents reflects the green technology conducive to saving resources, improving energy efficiency, preventing and controlling pollution, and achieving sustainable development. This data comes from the PatSnap database.
4. *Government support*. Attention allocation has a key role in managers’ limited rationality decision theory, and this indicator reflects the logical relationship between attention to green development and the government’s choice of carbon-reducing behavior, as measured by Nuo & Liu [82], which is statistically derived by local government reports combing key words such as low-carbon economy, green economy, eco-city, eco-civilization demonstration zone, and circular economy; The Survey and Evaluation Report 2020 on Online Government Services Capability of Provincial Governments and Key Cities (Good and Bad Evaluation of Government Services) compares and analyzes the overall situation of the current development of online government services and forms the integration index of government services in each province in terms of the effectiveness of online services, familiarity with online processing, completeness of service methods, coverage of service matters, and accuracy of office guidelines. The Chinese Government Transparency Index is one of the series of results in the Blue Book on the Rule of Law published by the Institute of Law of the Chinese Academy of Social Sciences, the Chinese Government Transparency Index Report (2019), which gives a comprehensive score to each provincial government’s openness in decision-making (35%), openness in management services (20%), openness in quality and results (25%), and construction of open government affairs platform (20%). (20%) for a total score.
5. *Economic development*. Provincial gross domestic product (GDP) and per capita gross regional product data are obtained from the National Bureau of Statistics. The digital financial inclusion index is obtained from the Peking University Digital Financial Inclusion Index, which contains comprehensive data on the breadth of digital financial coverage, the depth of digital financial use, and the degree of digitalization of financial inclusion.
6. *Demographic factors*. The selected provinces contain three indicators of year-end resident population, urbanization rate, and urban population density, which are all obtained from the National Bureau of Statistics of China.

### Research methodology

The hybrid approach of necessary condition analysis (NCA) and qualitative comparative analysis (QCA) is an emerging method to confirm necessary and sufficient causality. Whereas necessary condition causality refers to the fact that the outcome does not occur without a particular antecedent, sufficient condition causality refers to the fact that the antecedent (combination) sufficiently produces the outcome [83, 84]. It is possible to analyze how and to what degree antecedent conditions can serve as necessary conditions for the outcome using NCA, which specializes in analyzing necessary relationships [85]. In comparison, QCA adopts a comprehensive viewpoint and uses case-oriented comparative analysis [86] to pinpoint the causal links between conditional states and outcomes to respond to the query, "Which configurations of conditions lead to the desired outcome? Which configurationscause the result not to occur? Such inquiries.QCA concentrates on the analysis of "configuration effects," whereas conventional regression analysis adopts an atomic viewpoint and concentrates on the distinct "net effects" of individual variables [86]. Depending on the sort of variable, QCA is divided into three categories: csQCA (clear set qualitative comparative analysis), mvQCA (multivalued qualitative comparative analysis), and fsQCA (fuzzy set qualitative comparative analysis). Compared to csQCA and mvQCA, fsQCA has more flexible fixed distance and affiliation and greater benefits when managing qualitative data, limiting diversity, and streamlining configuration [87]. The article comes into this category and employs the fsQCA method to analyze the complex influence mechanism on carbon emission performance by the combination of elements influencing carbon emission performance forming various ecologies. The fsQCA method additionally combines the benefits of qualitative and quantitative analysis, providing answers to the "generalizability" of qualitative analysis in a few instances and partially making up for the limitations of large sample analysis for qualitative change and phenomenological analysis.

### Data calibration

Based on existing research theories, the article participates in the unified calibration of variables as fuzzy sets. According to the characteristics of the variable values, reference to the study [88] selected the 95% quantile, 5% fraction as set as a conditional variable calibration aiming point, representing fully affiliated, fully unaffiliated, selected 50% as the crossover point, and the larger the value of the outcome variable carbon emission intensity the lower the level of carbon emission performance it reflects, so the use of fully affiliated (5%), crossover point (50%), and The results of the calibration using full affiliation (5%), crossover (50%), and full disaffiliation (95%) are shown in Table 1:

**Table 1:** Calibration of variables.

Conditions and results	calibration		
	fully affiliated	intersection	fully unaffiliated
carbon emission performance	0.659	1.3285	6.2923
energy consumption	5.132999	2.962085	1.2390205
industrial structure	5.54406335	2.565259	1.8650337
technological innovation	5.859195	1.487705	0.72503925
government support	5.9754685	4.07558	2.3605885
economic development	5.0666528	1.571758	0.36427758
demographic factors	4.6511765	3.1122	1.910334

## Data analysis and empirical results

### Necessity analysis of individual results

NCA identifies whether an element is necessary for an outcome and analyzes how the necessary condition can affect the outcome. In NCA, the effect size (effect size) indicates the minimum level needed to produce a particular outcome from a necessary condition. It is generally considered that the effect size takes a value between 0 and 1, with larger representing a larger effect and less than 0.1 representing a too-low effect size [83]. The article uses ceiling regression (CR) and ceiling envelopment analysis (CE) to calculate the effect size of the 6-point antecedent variable. The results are shown in Table 2, and the necessary conditions in NCA method need to meet two conditions: the effect size (d) is not less than 0.1 [83], and the p-value showing the effect size is significant [84].

**Table 2:** Analysis results of necessary conditions of NCA method.

antecedent conditions	methods	C-accuracy	ceiling zone	Effect size (d)	Scope	P-value
energy consumption	CR	100%	0	0	0.96	1
	CE	100%	0	0	0.96	1
industrial structure	CR	70%	0.166	0.169	0.98	0.024
	CE	100%	0.146	0.149	0.98	0.009
technological innovation	CR	90.00%	0.137	0.148	0.92	0.009
	CE	100%	0.143	0.155	0.92	0
government support	CR	80.00%	0.221	0.237	0.93	0
	CE	100%	0.144	0.154	0.93	0
economic developmen	CR	83.30%	0.183	0.201	0.91	0.014
	CE	100%	0.185	0.203	0.91	0
demographic factors	CR	90%	0.025	0.027	0.93	0.34
	CE	100%	0.036	0.034	0.93	0.291

As a result, the effect sizes of industrial structure, technological innovation, government support, and economic development are more significant than 0.1. However, they are all less than 0.3, and their effect sizes are so small that they do not constitute the necessary conditions for the outcome.

In NCA, the term "bottleneck level" is equal to "effect size," and the term "bottleneck level (%)" denotes the percentage that must fall within the maximum observed range of the antecedent conditions in order to achieve a specific level of the maximum observed range of the results. According to Table 3,the bottleneck level analysis, it is clear that in order to achieve a 90% carbon emission performance level, there must be no bottleneck energy consumption conditions, a 60.7% level of industrial structure, a 53.5% level of technological innovation, a 70.2% level of government support, a 61% level of economic development, and a 7.7% level of demographic factors.

**Table 3:** NCA method bottleneck level (%) analysis results.

carbon emission performance	energy consumption	industrial structure	technological innovation	government support	economic development	demographic factors
0	NN	NN	NN	NN	NN	NN
10	NN	NN	NN	NN	NN	NN
20	NN	NN	NN	NN	NN	NN
30	NN	NN	NN	NN	NN	NN
40	NN	NN	NN	NN	NN	NN
50	NN	NN	NN	8.3	5	1.1
60	NN	4.8	4	23.8	19	2.8
70	NN	23.5	20.5	39.3	33	4.4
80	NN	42.1	37	54.8	47	6.1
90	NN	60.7	53.5	70.2	61	7.7
100	NN	79.3	70	85.7	75	9.4

Based on this, the NCA necessary condition results were validated using the fsQ CA method. The consistency evaluation of the antecedent variable on the result determines whether or not it is a necessary condition; if the consistency value is higher than 0.9, the antecedent variable is considered necessary for the result [89]. Table 4 displays the fsQCA results and the consistency of each condition’s necessity is <0.9. Consistent with the NCA results, no high carbon output performance creates the prerequisites for a high carbon performance.

**Table 4:** Necessity tests for individual conditions of the QCA method.

Condition Variables	High-level carbon emission performance		Non-high-level carbon emission performance	
	Consistency	Coverage	Consistency	Coverage
Energy consumption	0.485023	0.582699	0.886076	0.775087
Non-energy consumption	0.812788	0.907396	0.522943	0.42508
Industrial structure	0.652074	0.806268	0.590981	0.532051
Non-industrial structure	0.621544	0.676065	0.78481	0.621554
Technological innovation	0.663595	0.897196	0.398734	0.392523
Non-technological innovation	0.550691	0.55711	0.89557	0.659674
Government support	0.732143	0.868763	0.484177	0.418319
Non-government support	0.509793	0.575797	0.848101	0.697463
Economic development	0.718894	0.899784	0.422468	0.385004
Non-economic development	0.508641	0.547427	0.890032	0.697458
Demographic factors	0.602535	0.772526	0.49288	0.460118
Non-demographic factors	0.578917	0.610571	0.756329	0.580802

### Configuration analysis

**Fig 3:**
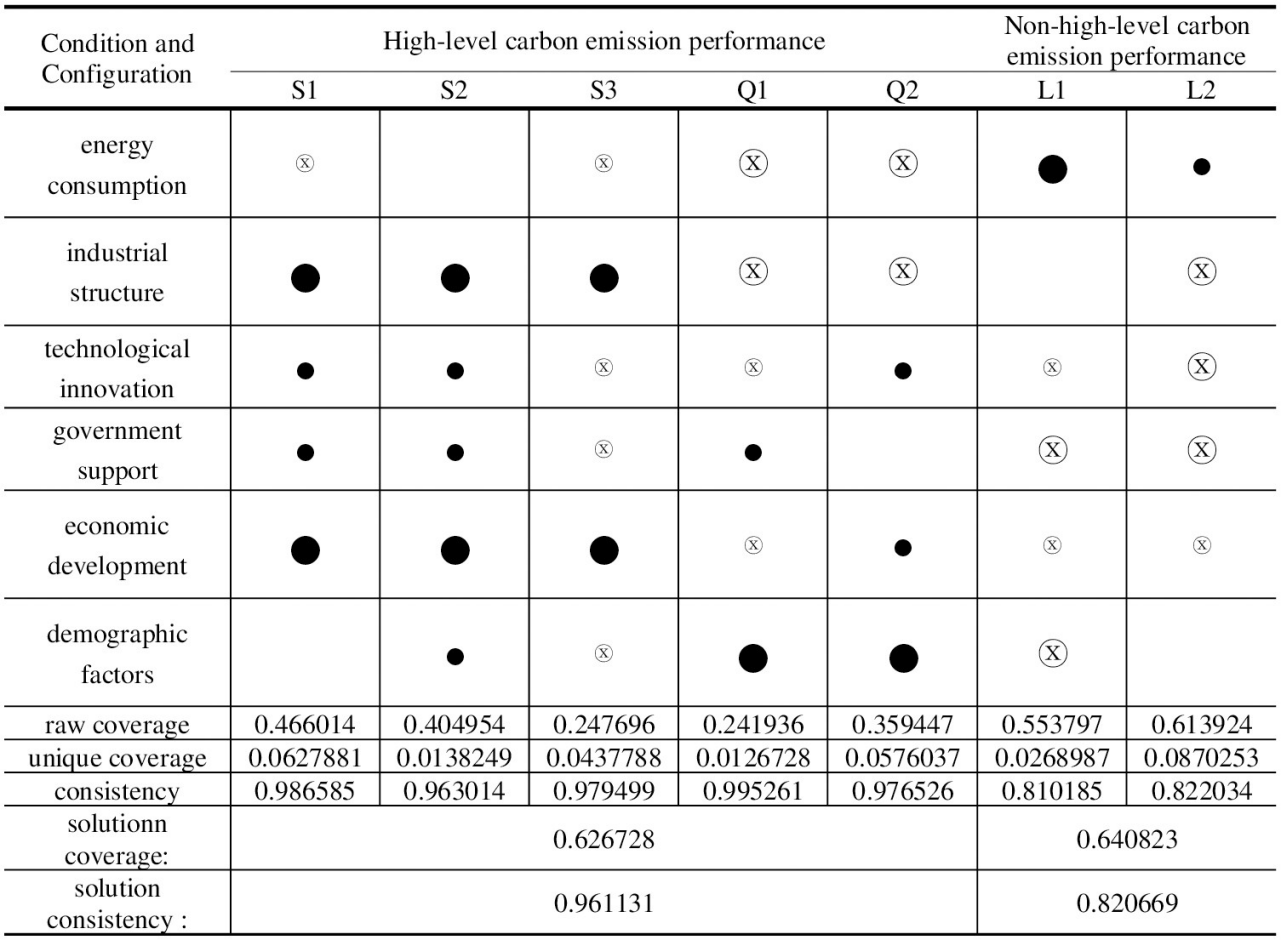
Configurations for achieving high/non-high carbon performance. Note: ● = core condition exists; ⓧ = missing core condition; ● = marginal condition exists; ⓧ = missing marginal condition.

After importing the calibrated data into the fsQCA3.0 software for processing, the original consistency threshold was set to 0.8, the frequency threshold to 1, and the PRI consistency threshold to 0.85 to construct the truth table, from which the final simple intermediate and complex solutions were obtained [87]. The core and marginal conditions are derived by nesting and contrasting the simple solutions, the intermediate solution that serves as the foundation for this article. A total of five high-carbon emission performance configurations and two non-high-carbon emission performance configurations are found. (as shown in Fig 3). The consistency level of the five positive single solutions (configurations) and the overall solution are better than the acceptable minimum standard of 0.75; the consistency and coverage of the overall solution are both 0.961 and 0.63, respectively. These five favourable configurations are believed to produce high-level carbon emission performance. Here, we divide the case provinces’ carbon emission performance into three echelons based on their rankings, with the first echelon being the top 25% of case provinces, the second echelon being 26%-50%, and the third echelon being 51%-100%. Generally, the typical provinces and cities of high-level performance cases appear in the top 50%. Since the high-level carbon emission performance configurations S1, S2, and S3 and configurations Q1 and Q2 have the same core conditions, according to the second-order equivalence, configurations have the same core conditions [90]. The core conditions for the S-configuration state are industrial structure and economic development, which can be further refined as multi-factor linkage under the dual-wheel drive of industrial structure and economic development. Depending on whether these conditions exist, the S-configuration state is further divided into full-factor-driven (S1, S2) and industry-economy dual-wheel-driven (S3) states. Under low energy consumption, the Q-configuration state is characterized as factor-driven and considers non-energy consumption as the core condition.

#### Multi-factor linkage driven by economic development and industrial structure

**Fig 4:**
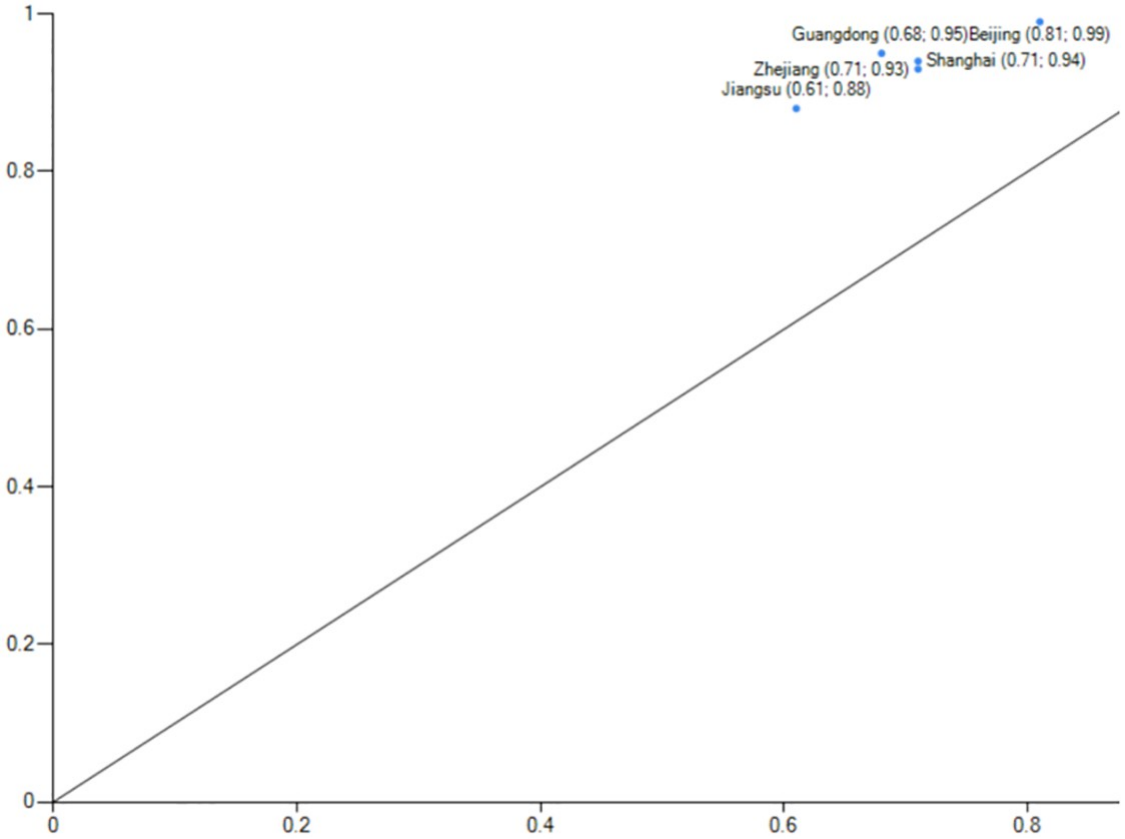
configuration S1 explain case.

**Fig 5:**
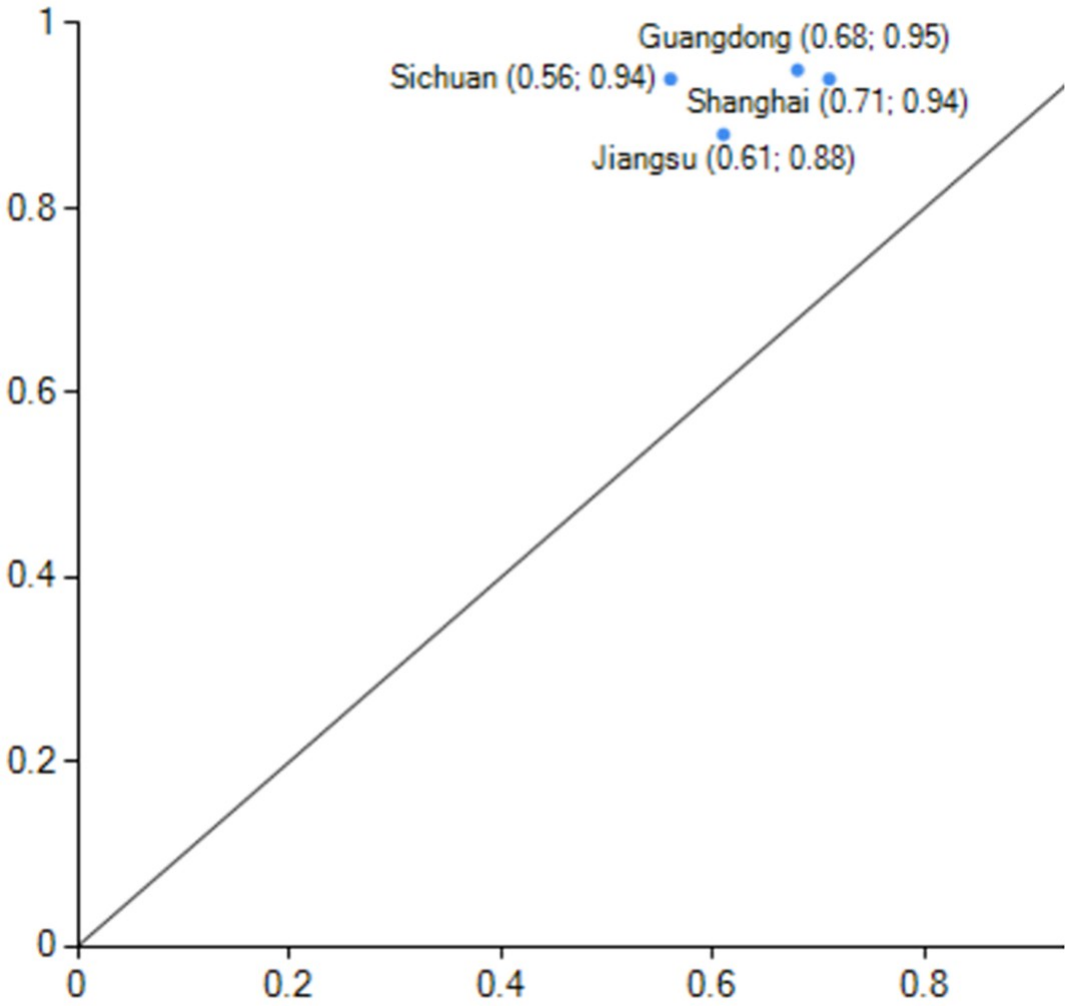
configuration S2 explain case.

*(1) Total factor-driven*. The consistency of configuration S1 is 0.986, and the original coverage is 0.466, indicating that about 46.6% of the cases of high-level carbon performance can be explained by configuration S1. The core of the configuration S2 is consistent with the core and marginal conditions of S1, with the difference that the demographic factor is present as a supplementary marginal condition in the S2 configuration, indicating that a higher level of carbon emission performance can be achieved with or without the presence of the demographic factor in the full factor-driven configuration. The consistency of configuration S2 is 0.986, and its initial coverage is 0.404, which explains 40.4% of the cases. The S1 and S2 configuration reveals that almost half of the cases belong to the all-factor-driven type.This type of region is dominated by economic development, with lower energy consumption, a rationalized and advanced industrial structure as the main features, technological innovation and government support as supporting factors, and other multi-factors synergistically contributing to a high level of carbon emission performance, regardless of whether the demographic factor is a supporting element or not.The typical case provinces for S1 and S2 are Beijing, Shanghai, Zhejiang, Guangdong, and Jiangsu(Figs 4 and 5).Except for Jiangsu Province, the typical case provinces belong to the first high-level carbon emission performance echelon. Shanghai has been actively supporting energy conservation and emission reduction measures as one of the first and second batches of national pilot cities for low-carbon provinces and cities. The Chinese government prioritizes low-carbon green development, and Shanghai is at the forefront of the country’s ability to deliver integrated government services and transparency. During the 12th and 13th Five-Year Plans, Shanghai achieved the national targets and went above and beyond them. Shanghai achieved and surpassed the national goals in the 12th and 13th Five-Year Plans. Shanghai has reduced its energy usage and CO2 emissions per unit of GDP by over 50% cumulatively since 2010. In addition to having a full variety of financial institutions and a complete financial infrastructure, Shanghai is a top-tier financial centre that has pioneered the introduction of important institutions and platforms in China and the development of green financial products. The development of many energy-saving and carbon-reduction-related innovations takes place in Shanghai, which is a regional centre for science and technology. A large overall impact of multi-factor synergy can be seen in the multi-factor drive’s high carbon emission performance level, primarily in the first echelon.

In particular, it should be noted that S1 and S2 have a high degree of similarity in terms of compositional conditions, and the only difference is that the population factor plays a role in the S2 grouping. The representative case provinces in the S2 grouping are Guangdong, Shanghai, Jiangsu, and Sichuan, while the case provinces and cities represented by S1 are Beijing, Guangdong, Shanghai, Zhejiang, and Jiangsu. Let us take S1 Beijing and Shanghai in S2 as an example. Both cities are municipalities directly under the central government. However, the total population and population density of Shanghai are more significant than that of Beijing, so when the population factor plays a role, Shanghai exists in S1 and S2 at the same time. When the population factor does not play a role, Shanghai exists in the S2 grouping state. The population factor is not a core condition of existence in both groupings. That is to say, in the case of economic development, when economic development and industrial structure exist as the core conditions and technological innovation and government support exist as the marginal conditions, some provinces and cities can achieve a high level of carbon emission performance regardless of whether the population factor plays a role or not.

**Fig 6:**
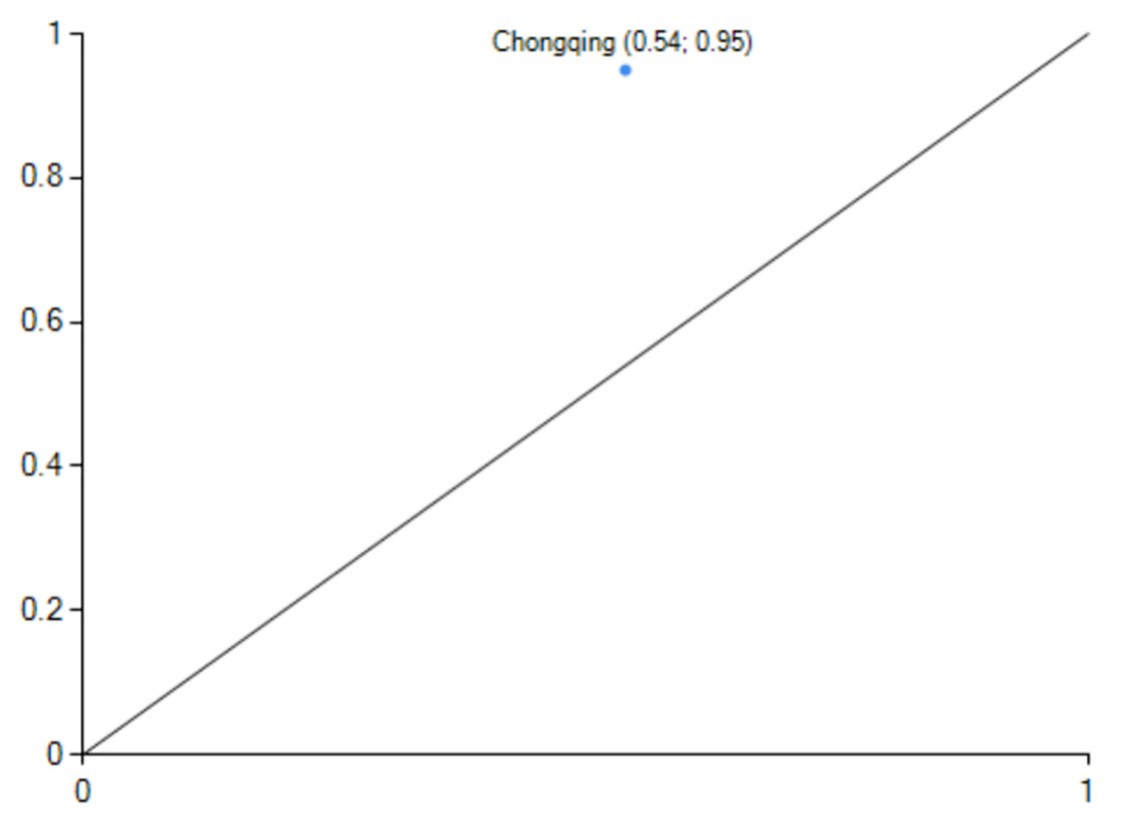
configuration S3 explain case.

*(2) The "industry-economy" two-wheel drive type*. Configuration S3 exists with industrial structure and economic development as the core conditions, non-energy consumption, non-technological innovation, non-governmental support and non-demographic factors as the marginal conditions, which can produce high-level carbon emission performance. The configuration consistency is 0.979, and the original coverage is 0.248. This path can explain about 24.8% of carbon emission performance cases. The configuration of the case, provinces and cities show that when the energy consumption is low, even if the technological innovation, government support and demographic factors are poor, with economic development as the traction, the rationalization of industrial structure and high-level can also produce a certain high level of carbon emissions performance. The typical case city of this configuration is Chongqing(Fig 6). In 2019, Chongqing’s per capita gross regional product exceeded US$10,000 (9th place), and the value added to the service industry grew by 6.4%, contributing 51.3% to economic growth. The industrial structure continues to be optimized, the pace of transformation and upgrading of traditional industries accelerates, and the added value of high-tech manufacturing and strategic emerging industries grows by 12.6% and 11.6%, respectively.

When we compare the S2 and S3 configurations, we can see that they share all of the same fundamental conditions but that the S3 configuration varies from the S2 configuration by not having any marginal conditions, such as technological advancement, governmental support, or demographic factors. The typical case provinces and cities’ carbon emission performance levels in the S2 and S3 histories are similar. However, the case provinces in S3 have lower carbon emission and economic growth levels than the typical case cities in S2. When the provinces in the S3 configuration increase their technological innovation and government support, they can further raise the region’s overall development level. However, they are unable to raise the carbon emission performance level significantly.

#### Factor-driven under low energy consumption

**Fig 7:**
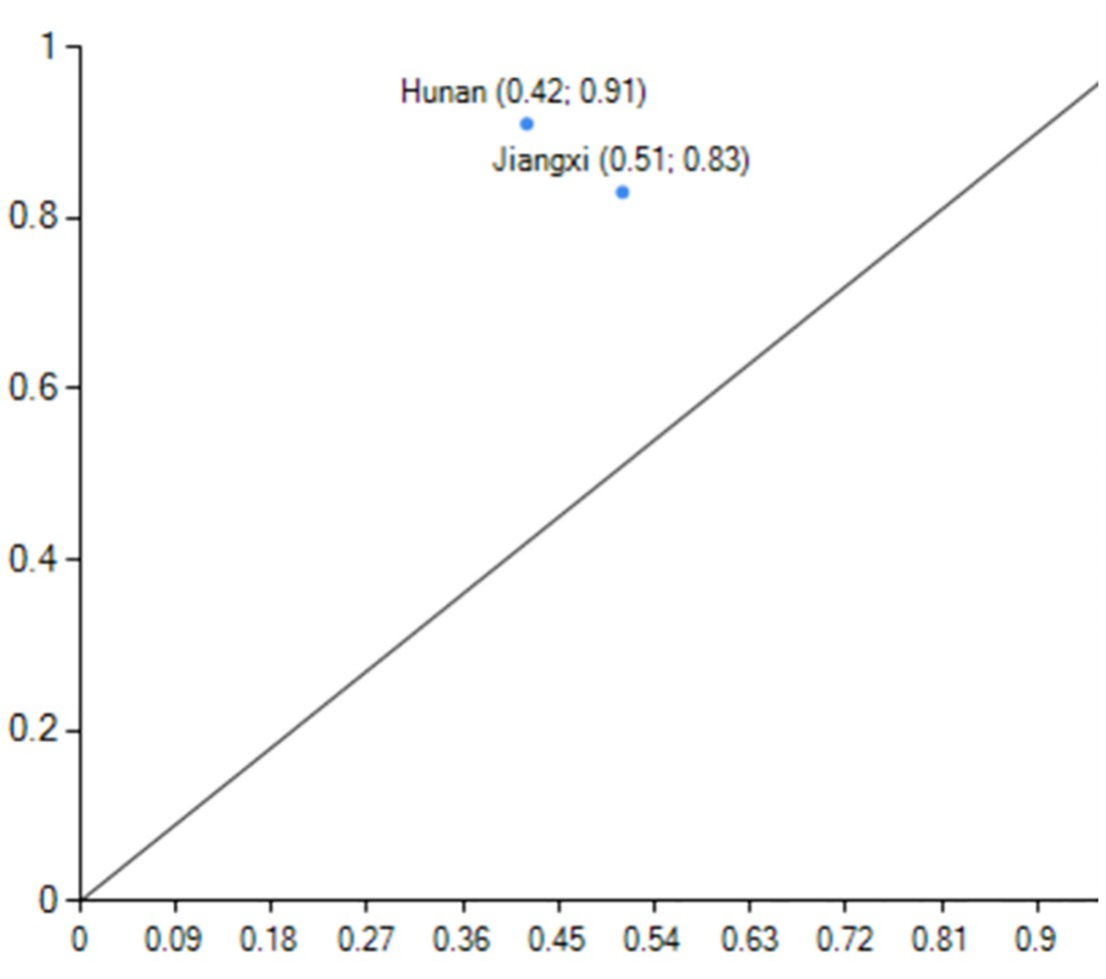
configuration Q1 explain case.

**Fig 8:**
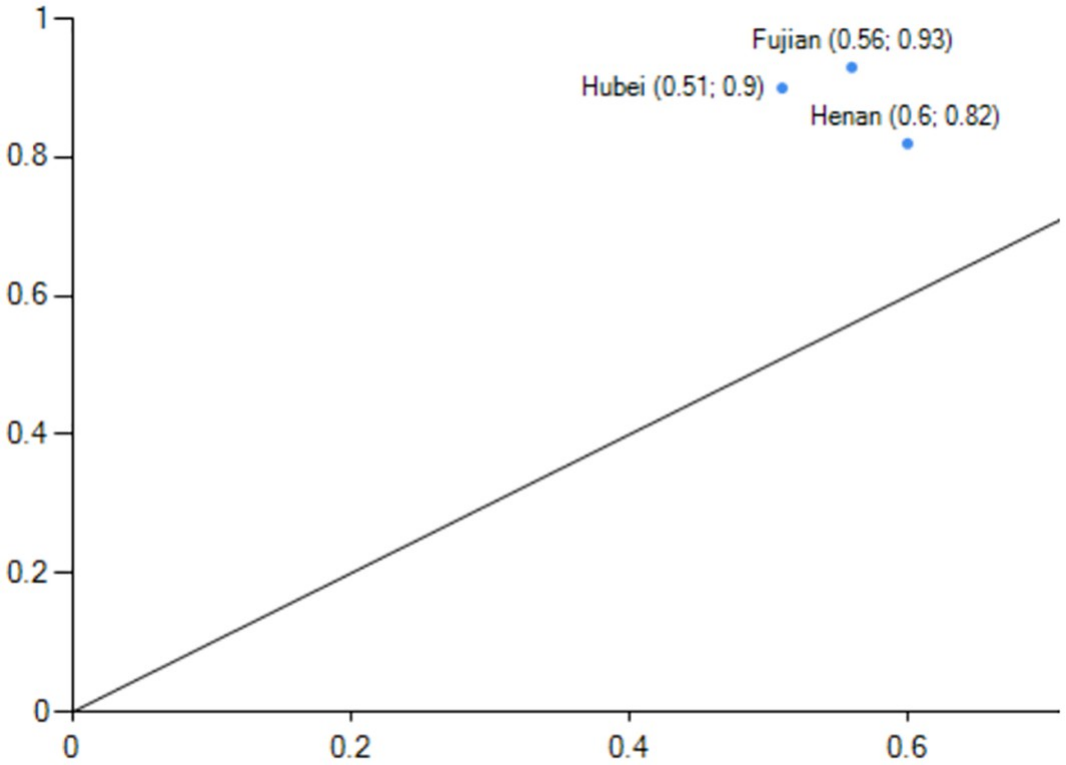
configuration Q2 explain case.

Configurations Q1 represents the high carbon emission performance level caused by non-energy consumption and demographic factors as core conditions and government support, non-industrial structure, non-technological innovation, and non-economic development as marginal conditions. With a consistency of 0.931 and original coverage of 0.241, this pathway can account for about 24.1% of carbon emission performance cases. With a consistency of 0.976 and original coverage of 0.359, Configurations Q2 represents the high carbon emission performance caused by non-energy consumption as a core condition and technological innovation, economic development, demographic factors, and non-industrial structure as marginal conditions. This pathway can account for about 35.9% of carbon emission performance cases. The marginal conditions of the Q1 and Q2 configuration have a substitution relationship, and the Q configuration demonstrates that even in regions where industrial structure and economic development are not core strengths, a certain high level of carbon performance can be achieved through government support and demographic factors and technological innovation to make up for it. Jiangxi,Hunan are the sample cases province for the Q1 configuration, and Henan, Fujian(Fig 7), and Hubei are the representative cases cities for Q2(Fig 8). Jiangxi and Henan are in the second echelon of high-level carbon emission results, while Fujian,Hunan and Hubei are in the first. Using Henan Province as an example, Henan Province has a strong economic development momentum in 2019 (fifth place in GDP), a certain level of science and technology innovation (ninth place), and a population that can provide enough labour for economic development. Henan Province is also China’s third most populous province, which can promote economic growth, reduce carbon emission intensity, and improve carbon emission performance.

### Configuration analysis of non-high-level carbon emission performance

The two configurations, L1 and L2 that generate non-high-level carbon emission performance can be further analyzed to verify the causal asymmetry. High-level carbon emission performance can only be accomplished with excessive energy consumption, an unfavourable industrial structure, a lack of technological innovation, inadequate government support, and an unfavourable population composition.

## Differentiation path of carbon emission performance in eastern, central and western China

**Fig 9:**
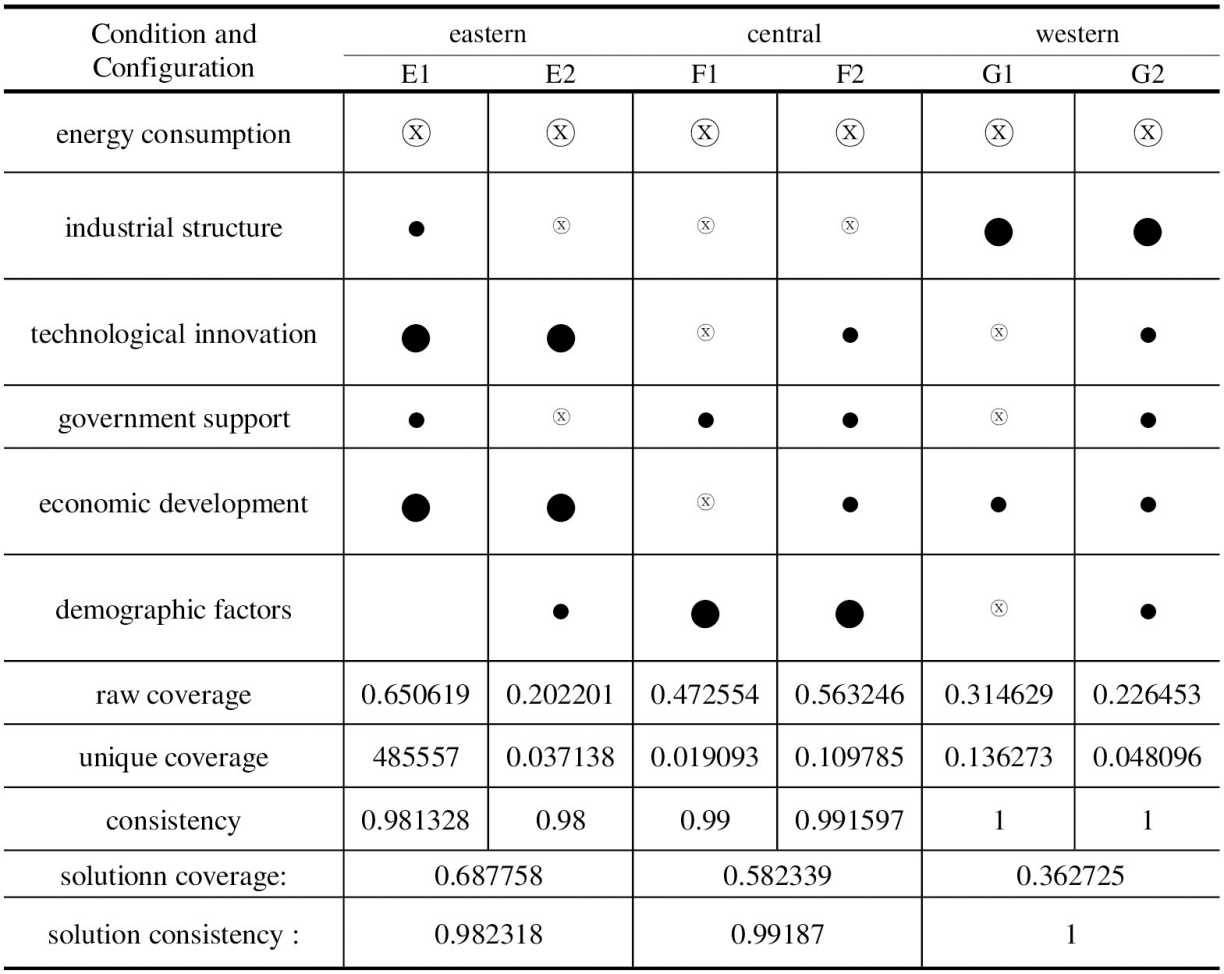
Configuration analysis of carbon emission performance (high level) in eastern, central and western regions. Note: ● = core condition exists; ⓧ = missing core condition; ● = marginal condition exists; ⓧ = missing marginal condition.

Local policies have obvious heterogeneity because of the level of economic development, resource endowment, and natural environment in different regions of China. The performance of carbon emissions is differentiated by energy consumption, industrial structure, technological innovation, economic growth, and demographic factors. According to the division of the National Bureau of Statistics for economic zones, we classify the sample provinces and cities according to four regions: central, east, west, and northeast, and because only three cases in the northeast could not derive practical configuration effects, they were excluded in this part, and the remaining 27 provinces and cities were analyzed by fsqca3.0 to obtain the configuration results of high carbon emission performance in each region as shown Fig 9.

In eastern China, there are two configurations of high-level carbon emissions, E1 and E2, and the main drivers of both are non-energy consumption, technological innovation, and economic growth. It shows that both configurations depend on a particular degree of economic development and that the fundamental conditions of technological innovation and economic growth have a greater impact on the factors determining high-level carbon emission performance. The multi-factor synergistic configuration E1 can accomplish a higher degree of carbon emission performance than configuration E2, which needs industrial structure and government support when the two configurations are compared. E Configuration demonstrates how technological innovation has altered the local development paradigm and can successfully alter the kinds of sectors with high energy consumption to achieve low-carbon, coordinated, sustainable development. The eastern region of China has assumed the lead in the economy and possesses significant scientific and technological power after more than 40 years of reform and opening up. Shanghai, Beijing, Tianjin, Guangdong, Jiangsu, Zhejiang, and other eastern provinces have always belonged to the first echelon in terms of science and technology innovation level, and the eastern region has absolute advantages in innovation advantages and leading development, according to the multi-year report "China Regional Science and Technology Innovation Evaluation Report." Innovation in science and technology is a significant internal factor that supports the upgrading and optimizing of the industrial structure and raises the standard of economic growth. The eastern region accelerates the development of new-generation technology sectors like digitalization and intelligence based on bolstering science and technology innovation. It strengthens the framework for developing a low-carbon and green regional economy.

In the central area, two configurations work well regarding high carbon emissions: F1 and F2, which have demographic factors and non-energy consumption as their fundamental conditions. A certain degree of carbon emission performance can be attained in the F1 configuration when governmental support is strong, and the population factor has a particular impact. The F2 configuration demonstrates that, even in regions lacking an advantageous industrial structure, a certain level of economic development can result in high carbon emission performance by relying on specific technological innovation capabilities and demographic factors. The economic growth of China is centred in the central region. Although the benefits of technological innovation, industrial transformation, and government support are only sometimes apparent, total market vitality is high, economic growth is rapid, and green development has significant room for expansion.

The low energy consumption and industrial structure underpin the Western region’s G1 and G2 configurations of high carbon emission performance. Sichuan and Chongqing are the usual representative cities of high-level performance in G configuration. High-level carbon emission performance is discovered to be concentrated in the southwest area of the western region. The area has a solid economic base but needs fundamental advantages in government support and technological innovation, and its energy consumption could be better. The western region can take on the industrial transformation of the eastern region, optimize and improve the design of high-tech sectors, and improve carbon emission performance.

In the regional comparison, it is also discovered that there are similarities and differences in the driving paths of high carbon emission performance in eastern, central, and western China. Both eastern, central, and western regions can exhibit high carbon emission performance when all factors are driven simultaneously. However, the conditions for high carbon emission performance are noticeably different when limited by the resource endowment conditions of each region. The central region prefers government support and demographic factors, the western region prefers upgrading industrial structures based on a certain level of economic growth, and the eastern region emphasizes the benefits of economic and technological innovation. A thorough examination of the mechanisms underlying each pathway’s formation can aid in exploring the choice of low-carbon development in China and explain the distinct properties of common and unique influencing factors resulting from "different pathways" of high-carbon emission conditions.

## Robustness test

This paper conducts robustness tests on the antecedent configuration of high-level carbon performance. First, the case number threshold is raised from 1 to 2, and the two configurations generated are consistent with two existing histories. Next, the PRI consistency is raised from 0.85 to 0.9 to include four histories in the existing histories. Finally, the calibration anchor points are raised, and the fully affiliated and fully unaffiliated anchor points are raised to a level consistent with the fully affiliated and unaffiliated histories. Fourth, the four municipalities of Beijing, Shanghai, Tianjin, and Chongqing are deleted, and obtain three high-level carbon emission performance configurations in the model. The 85th percentile, 15th percentile, and 50th percentile of the crossover point remain unchanged, the case threshold is 1, the original consistency is 0.8, and the PRI is 0.8. In conclusion, the findings of high-level carbon emission performance are reliable.

## Conclusion and discussion

Existing studies have pointed out that improving carbon emission performance can promote coordinated development between economic development and environmental governance. Studies on carbon emission performance have been able to show the temporal and spatial evolution of carbon emission performance from various regional levels and have also explored the complex mechanism of the main influencing factors affecting carbon emission performance comprehensively from various dimensions of development. However, previous studies tend to pay too much attention to the individual effects of the main influencing factors or the mediating effects among multiple influencing factors but have not explored the interdependence of the elements affecting carbon emission performance from the perspective of complex systems, which may evolve different ecosystems and form the related problems of multiple equilibria. The systematic differences for different provinces and cities may lead to differences in carbon emission and economic development enhancement, forming multiple paths for developing different carbon emission performances. Therefore, this paper adopts a group perspective from the social, economic, political, and other perspectives, constructs a six-dimensional mechanism framework affecting carbon emission performance, systematically analyses how the coupling between the elements affecting carbon emission performance can adequately promote economic development and reduce carbon emissions, and then puts forward the path to effectively improve carbon emission performance, which is of particular reference value for the study of the theoretical mechanism of perfecting carbon emission performance and improving the government’s practice of carbon emission performance. It has a specific reference value for improving the theoretical mechanism of carbon emission performance and enhancing the government’s carbon emission performance practice.

This study examines the conditional configuration and driving path of high-level carbon emission performance under the synergistic effect of multi-dimensional conditions using fsQCA, NCA, and other methods based on a system-wide perspective. It then answers the key components and intricate linkage mechanisms that, to a certain extent, influence regional governments’ carbon emission performance levels. The following are the major conclusions:(1) Configuration diversity argues that there are multiple ways to choose the best route for high-level performance. High-level performance only requires a few prerequisites to be met. According to multi-factor synergy, high-level success can be attained in "different ways" when combined with core and marginal conditions. The regional configurations with economic growth as the dominant group and the regional configuration with low energy consumption as the dominant advantage can generate high-level carbon emission performance in this article’s two second-order equivalent configurations of S and Q. (2) Discrimination by region. The driving path of high-level carbon emission performance varies and has similarities in China’s eastern, central, and western areas. The total factor driving model applies to the eastern, central, and western regions, but due to varying configuration conditions, each region emphasizes key advantages differently. It further describes that the multi-factor driving path is grounded in reality and tailored to local circumstances by corresponding to the differentiation of typical cities in each configuration.

Although there are differences between this paper and previous studies in terms of research methodology, it is undeniable that the influencing factors of carbon emission performance selected by the author are based on what has been fully demonstrated and explored by previous authors. The good side is that we have fully affirmed the previous scholars’ research on the influencing mechanism of these elements. Based on this, we have constructed a research framework that belongs to this paper. However, regrettably, the article cannot again validate the previous research on the effects of these elements on carbon emission performance. The article, unfortunately, could not re-validate the previous research on the effect of these elements on carbon emission performance.

Nonetheless, our research results can still echo the results of previous studies in some points. For example, the S2 and S3 groupings are entirely complementary, and comparing the S2 and S3 groupings, we can find that they have the same core conditions but opposite conditions regarding technological innovation, governmental support, and demographic factors. The carbon emission performance levels of the typical case provinces and cities in the S2 and S3 groupings are comparable. However, the carbon emissions of the case provinces in the S3 grouping are lower than those of the typical case cities in the S2 grouping regarding the level of carbon emissions, economic development, and the degree of industrial transformation. So we think that the gap may be related to government support and technological innovation, which can be echoed with the previous study that technological innovation can indirectly reduce carbon intensity by promoting the upgrading and optimization of industrial structure [91] and that the regions with weakened government support are also detrimental to regional economic development [92].

The research method still has some limitations, which merit more investigation. First, this study only chooses the cross-sectional data from 2019 for analysis due to current data availability. The research’s dynamic trend needs to be adequately reflected in the findings, and the driving mechanism affecting carbon emission performance needs further exploration. Second, the research uses second-hand public data only discussed from the macro level and fails to reveal the impact mechanism behind carbon emissions from the micro level, even though the conditional factors affecting carbon emission performance include many variables that have been tested. Given the author’s chosen provincial-level indicators, the differences below the provincial level need to be further investigated.

## Supporting information

S1 Data (XLSX)
